# Air Stable Nickel-Decorated Black Phosphorus and Its Room-Temperature Chemiresistive Gas Sensor Capabilities

**DOI:** 10.1021/acsami.1c10763

**Published:** 2021-09-10

**Authors:** Matteo Valt, Maria Caporali, Barbara Fabbri, Andrea Gaiardo, Soufiane Krik, Erica Iacob, Lia Vanzetti, Cesare Malagù, Martina Banchelli, Cristiano D’Andrea, Manuel Serrano-Ruiz, Matteo Vanni, Maurizio Peruzzini, Vincenzo Guidi

**Affiliations:** † Department of Physics and Earth Sciences, University of Ferrara, Via G. Saragat 1/C, Ferrara 44122, Italy; ‡ Italian National Council for Research - Institute for the Chemistry of OrganoMetallic Compounds (CNR ICCOM), Via Madonna del Piano 10, Sesto Fiorentino 50019, Italy; § MNF - Micro Nano Facility Unit, Sensors and Devices Center, Bruno Kessler Foundation, Via Sommarive 18, Trento 38123, Italy; ∥ Italian National Council for Research, Institute of Applied Physics “Nello Carrara”, Via Madonna del Piano 10, Sesto Fiorentino 50019, Italy

**Keywords:** black phosphorus, nickel, nitrogen dioxide, gas sensors, ambient stability

## Abstract

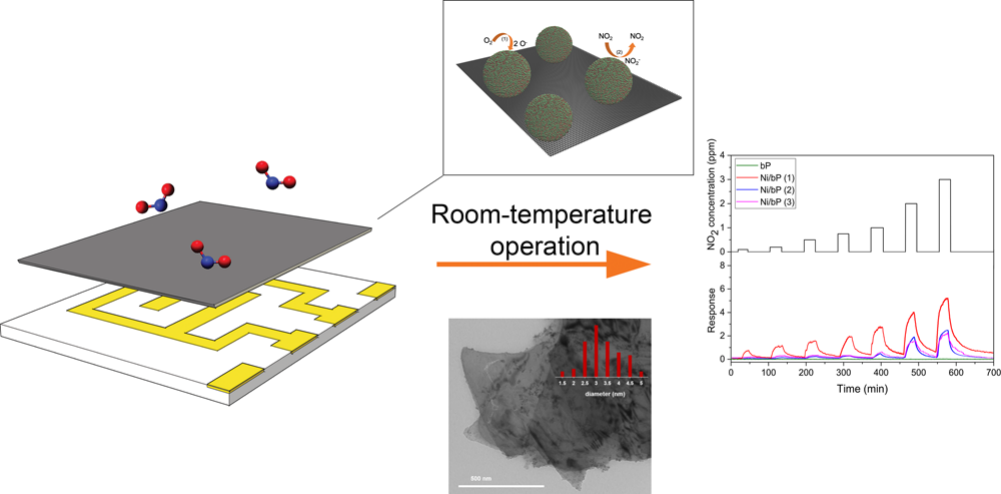

In the rapidly emerging field of layered two-dimensional functional materials, black phosphorus, the P-counterpart of graphene, is a potential candidate for various applications, e.g., nanoscale optoelectronics, rechargeable ion batteries, electrocatalysts, thermoelectrics, solar cells, and sensors. Black phosphorus has shown superior chemical sensing performance; in particular, it is selective for the detection of NO_2_, an environmental toxic gas, for which black phosphorus has highlighted high sensitivity at a ppb level. In this work, by applying a multiscale characterization approach, we demonstrated a stability and functionality improvement of nickel-decorated black phosphorus films for gas sensing prepared by a simple, reproducible, and affordable deposition technique. Furthermore, we studied the electrical behavior of these films once implemented as functional layers in gas sensors by exposing them to different gaseous compounds and under different relative humidity conditions. Finally, the influence on sensing performance of nickel nanoparticle dimensions and concentration correlated to the decoration technique and film thickness was investigated.

## Introduction

The increasing demand of new materials in the field of gas sensing has constantly pushed forward the research in materials science and technology in recent years. In particular, the needs of highly selective materials over a single analyte together with room-temperature (RT) operation are key features to possess for any high-performance gas sensing device. In the majority of currently employed materials for gas detection, such as the most used metal-oxide semiconductors (MOXs), the lack of selectivity and the need of high operating temperature (ranging within 200–650 °C) are well-known drawbacks that prevent the wide use of MOXs in harsh or industrial environments.^1−4^ At room/low temperature, MOX sensors lack sensitivity, stability, and ultimately reversibility of the detection process at the sensing film surface. Lately, two-dimensional (2D) materials such as graphene, transition metal dichalcogenides (TMD), and phosphorene have attracted the interest of the research community due to unique chemical and physical characteristics, showing their room/low-temperature kinetics of reaction as rapid as those for thermo-activated conventional material-based devices, though their stability still remains significantly lower.^5^ In fact, the atomically thin geometry makes their electronic properties highly susceptible to the environmental changes.

Exfoliated black phosphorus (bP) is particularly interesting for use in electronic sensing devices, not only for its unique direct and tunable band gap ranging from 0.3 eV (bulk) to 2.0 eV (monolayer) and its high carrier mobility^6^ (up to 6000 cm^2^ V^–1^ s^–1^) but also for its high chemical adsorption energy, less out-of-carrier conductance, and large availability of adsorption sites caused by its corrugated surface structure.^7^ Comparing the three main allotropes (white, red, and black) of phosphorus, the bP has diverse qualities including thermodynamic stability, insolubility in most solvents, lower chemical reactivity, and nonflammability. Recent density functional theory (DFT) studies carried out on the molecular adsorption energy of bP have suggested its use as a high-performance chemical sensor, confirming its higher adsorption energy, even higher than other 2D materials, such as graphene and transition metal dichalcogenides.^8−11^ Noteworthily, bP showed good performances as a humidity sensor^12,13^ and also exhibited selectivity for the detection of NO_2_, featuring high sensitivity and a limit of detection (LOD) of parts per billion (ppb).^14,15^ Despite this, ambient stability in air remains an issue that prevent the practical uses of bP-based gas sensors. In fact, few research works have been dedicated to the development of suitable passivation strategies able to preserve the intrinsic properties and sustain the long-term stability of the material.^16,17^

In order to achieve this goal and further improve the performance of the bP-based sensing devices, *n*- or *p*-type functionalization with different chemical species^18,19^ can be exploited to tune material properties, as already proven for other 2D sensing materials such as graphene and reduced graphene oxide.^20−23^ If the concentration and chemical sensitization levels are efficiently controlled, the selective analysis of target chemicals can be achieved.^24,25^ Commonly, the sensitivity of a *p*-type material can be enhanced by tuning the morphology of the nanostructures,^26^ doping with additives to electronically sensitize the oxide semiconductor,^27^ or loading with noble metals^25^ to chemically sensitize the active surface. Among these methods, decoration with additives can modulate the concentration of charge carriers, which may have a subsequent effect on sensitivity.

With the aid of first-principles calculations, a systematic study on the binding energy, geometry, magnetic moment, and electronic structure of several metal adatoms adsorbed on phosphorene has been carried out, predicting that the immobilization of transition metals on the surface of phosphorene is feasible while preserving its structural integrity.^28−31^ Despite the importance of adopting a customized decorating strategy, few materials such as Au,^15^ Pt,^25^ CdS,^32^ TiO_2_,^27^ SrTiO_3_,^33^ Cs_2_CO_3_ and MoO_3,_^24^ and ZnO^17^ have been incorporated into bP to improve its sensing performance. However, research has remained confined to the investigation of the carrier concentration and the *n*- or *p*-decorating level of bP with a simple device structure.

The effects of decoration on the chemiresistive gas sensing capability of a bP system have not been widely investigated so far. Toward this direction, nickel is a promising candidate for bP functionalization since its capability to improve bP ambient stability has been demonstrated.^34^ Moreover, nickel is well-known for its gas sensing potential in various structures and under diverse conditions.^26,35−37^ In addition, a high adsorption energy (*E*_ads_) of −4.09 eV^28^ for Ni-decorated bP was obtained by first-principles calculations. This value is much larger than the corresponding *E*_ads_ of Ni adatoms on graphene,^38^ suggesting a more effective functionalization.

In this work, by applying a multiscale characterization approach, from ab-initio simulation to morphological, chemical, structural, and electrical characterization, we demonstrated the enhanced ambient stability in air and functionality improvement of nickel-decorated bP (Ni/bP) films for gas sensing prepared by a simple, reproducible, and affordable deposition technique. Furthermore, we studied the electrical behavior of these films aiming to their application as functional layers for gas sensing by exposing them to different gaseous compounds (NO_2_, CO_2_, H_2_, NH_3_, CO, benzene, ethanol, ethylene, formaldehyde, H_2_S, and SO_2_) and to different relative humidity (RH%) conditions. Moreover, the influence on sensing performance of nickel nanoparticle (Ni NP) dimensions and concentration related to the decoration technique and the film thickness was investigated. In particular, the sensing performance of the films was studied in room-temperature operation mode to highlight possible technological advantages for this novel application of Ni/bP films.

## Experimental Section

### Chemicals

All manipulations related to the synthesis of the nanomaterials were performed under an inert atmosphere using Schlenk techniques. Tetrahydrofuran (THF) was distilled from sodium/benzophenone and degassed prior to use. Methanol was distilled from Mg/I_2_. Dry dimethysulfoxide (DMSO) was purchased from Sigma Aldrich.

### Synthesis of Bulk Black Phosphorus and Liquid Phase Exfoliation

Crystals of black phosphorus were prepared according to a previously published procedure.^39^ The preparation of bP nanosheets was carried out by liquid phase exfoliation. bP microcrystals (5 mg) were suspended in 5 mL of dry DMSO, and 3–5 μL of degassed water was added. The vessel was closed under nitrogen, and it was kept for 5 days under the action of ultrasounds in a sonicator bath set at a frequency of 37 KHz and at 30 °C.^40^ The bP nanosheets were collected by centrifugation and washed with ethanol and acetone. Finally, they were suspended in dry THF (4.0 mg/1.0 mL) and used as a reference in the device described below.

### Synthesis of Ni/bP (**1**) and Ni/bP (**1a**)

#### Preformed Nickel Nanoparticles with an Average Diameter of 11.9 nm Deposited on bP

Preformed Ni NPs were synthesized as reported in the literature^41^ and afterward were immobilized on bP. To a freshly prepared suspension of few-layer bP in dry THF (3.0 mg bP, 3.0 mL), a black colloidal solution of nickel nanoparticles dispersed in dry THF (0.800 mL, 0.0175 M) was added dropwise under vigorous stirring at room temperature in an inert atmosphere. After stirring for 30 min, degassed acetone (10.0 mL) was added and the mixture was centrifuged at 9000 rpm for 20 min. The black residue was washed once more with acetone (10.0 mL), dried under a stream of nitrogen, and resuspended in THF.

The sample was analyzed by inductively coupled plasma mass spectrometry (ICP-MS), with results of a final molar ratio P:Ni = 10, and was named Ni/bP (**1**); the sample Ni/bP (**1a**) having P:Ni = 3 was prepared in a similar way.

### Synthesis of Ni/bP (**2**)

#### Nickel Nanoparticles with an Average Diameter of 3.0 nm Grown *In Situ* on bP

To a suspension of 2D bP (5.0 mg, 0.161 mmol) in 6.0 mL of dry THF, 40 mL of dry methanol was added. The suspension was stirred at RT for 10 min; after, a solution of NiCl_2_·6H_2_O (5.1 mg, 0.0214 mmol) in 255 μL of methanol was added and the resulting mixture was stirred for 10 min. Afterward, the addition of NaBH_4_ as a solid (10.0 mg, 0.265 mmol) changed the suspension from gray to black and stirring was kept at the maximum speed for 10 min. At this point, the mixture was centrifuged (9000 rpm for 20 min), the supernatant was discarded, and to the black residue, methanol (10.0 mL) and THF (2.0 mL) were added to wash it by another centrifugation cycle. The resulting black solid was dried under vacuum. From ICP-MS analysis, results show a final molar ratio P:Ni = 10.

### Synthesis of Ni/bP (**3**) and Ni/bP (**3a**)

#### Preformed Nickel Nanoparticles with an Average Diameter of 4.5 nm Deposited on bP

The synthesis of preformed Ni NPs having an average diameter of 4.5 nm was carried out according to a slightly modified published procedure.^42^ To solid Ni(acac)_2_ (50.0 mg, 0.194 mmol) was added oleylamine (510 μL, 1.557 mmol), and the mixture was degassed and then heated in a closed Schlenk up to 115 °C. The hot mixture, of light blue color, was quickly transferred by a syringe to another Schlenk containing trioctylphosphine (TOP, 870 μL, 1.946 mmol) warmed up to 220 °C. The final reaction mixture quickly became black and was kept at 220 °C for 30 min under vigorous stirring. After cooling down to RT, degassed acetone (10.0 mL) and ethanol (10.0 mL) were added and the suspension was put in a freezer (−20 °C) to precipitate the Ni NPs. The supernatant was discarded, and the black solid was washed once again with ethanol and dried under vacuum. Ni NPs were suspended in 10 mL of dry toluene, and the concentration of the stock solution was asserted by ICP-MS analysis to be 5.38 × 10^–3^ M.

The nanohybrid Ni/bP (**3**) was prepared by immobilization of the above-described Ni NPs on exfoliated bP following a procedure similar to the one used for Ni/bP (**1**). To a freshly prepared suspension of few-layer bP (5.0 mg, 0.161 mmol) in 20 mL of dry THF, a colloidal solution of the above-prepared Ni NPs (5.5 mL, 0.0295 mmol) was added dropwise under nitrogen at room temperature. After stirring for 3.5 h, the mixture was centrifuged at 9400 rpm for 20 min. The brown supernatant was discarded, and the black residue was washed once more with THF (15 mL). The black residue was dried under vacuum and named Ni/bP (**3**). Afterward, the same procedure was repeated but with a reduced reaction time (only 1 h) and the sample was named Ni/bP (**3a**). From ICP analysis, results show a final molar ratio of P:Ni = 10 for Ni/bP (**3**) and P: Ni = 20 Ni/bP (**3a**).

Samples were characterized by transmission electron microscopy (TEM), X-ray photoelectron spectroscopy (XPS), powder X-ray diffraction (PXRD), and Raman spectroscopy. (Additional details and characterizations can be found in the Supporting Information).

### Sensor Fabrication Method

**Figure 1:**
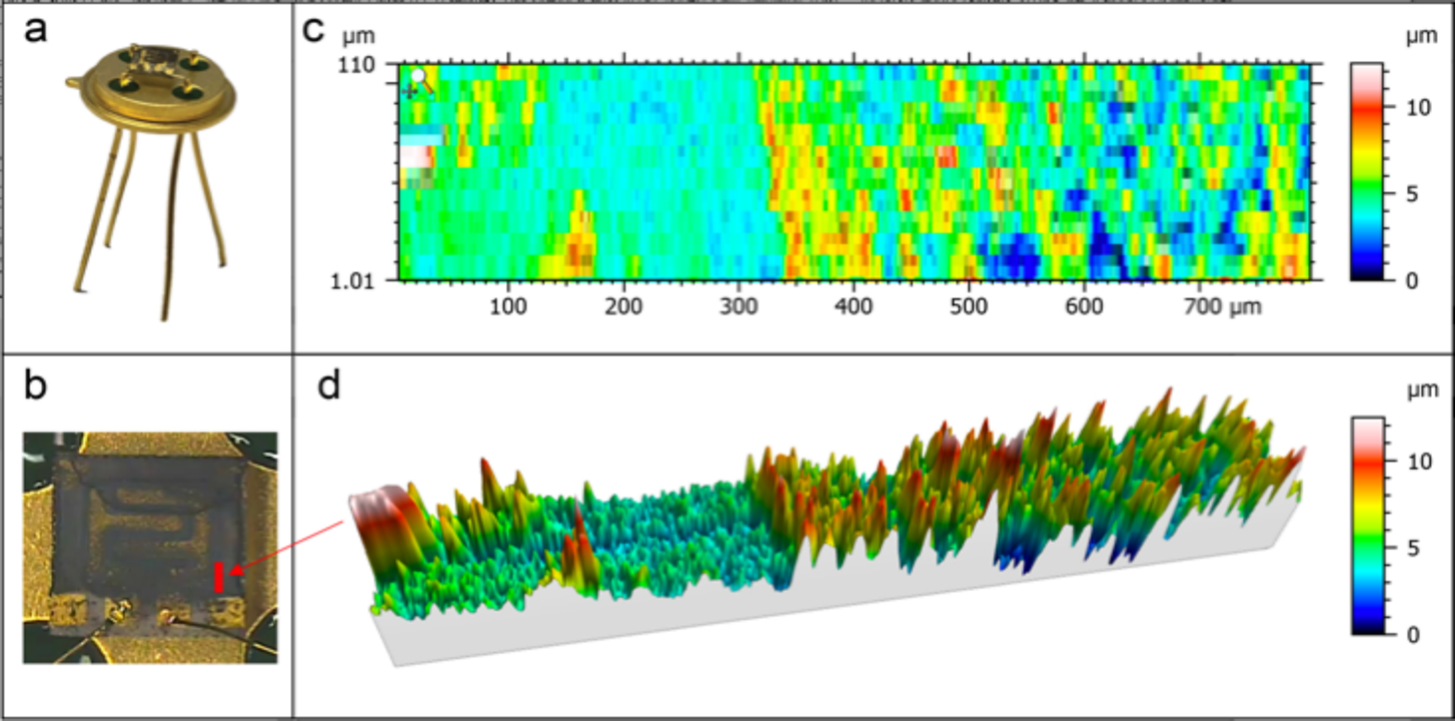
Photos of the (a) final sensing device and (b) film deposited on a substrate. (c) 2D and (d) 3D false color view of the material surface with the thickness information obtained with the KLA-Tencor P-6 stylus profilometer.

Six samples in a suspension of THF, all with the same concentration (5.0 mg in 1.25 mL), were used to prepare the following films: pristine 2D bP, Ni/bP (**1**), Ni/bP (**1a**) Ni/bP (**2**), Ni/bP (**3**), and Ni/bP (**3a**). The sensing films were prepared by dropping 50 μL of each solution on Al_2_O_3_ substrates with built-in interdigitated gold electrodes followed by spin-coating (at 3000 rpm for 20 s) and then drying at RT for 15 min. The deposition was repeated 4, 8, and 16 times in order to obtain three different films with average thicknesses of 2, 4, and 10 μm, respectively. A pristine bP-based film was prepared only with an intermediate thickness of 4 μm. The film thickness was measured by means of a KLA-Tencor P-6 stylus profilometer. The sensor substrate was subsequently packaged on a commercially available TO-39 support via thermo-compression bonding for electrical characterization (see Figure 1).

### Electrical Characterization

In order to investigate the potential sensing properties of Ni/bP-based devices, diverse electrical conductance measurements were performed at room temperature (25 ± 2 °C) by exposing the sensing films to controlled gaseous mixtures. In particular, synthetic air (20% O_2_ and 80% N_2_) and target gases were fluxed and mixed from certified cylinders (N 5.0 degree of purity) through mass-flow controllers, achieving a total flow of 500 sccm.

The sensors were electrically characterized by using a dedicated apparatus, which consists of a custom-made gas-flow test chamber (2000 cm^3^) managed by a suitable data acquisition system (see Figure S1, Supporting Information). A constant bias of 5 V was applied to the two interdigitated gold electrodes, and the sensing signal, i.e., the change in the electrical conductance of the sensor upon exposure to the analytes, was monitored and acquired. The acquisition circuit is based on an operational amplifier (OA). The voltage values *V*_in_ and *V*_out_ are connected at the ends of the sensor resistor *R*_s_ and applied load resistor *R*_f_, respectively. Then, the gain is given by *V*_out_/*V*_in_ = – *R*_f_/*R*_s_. The expression for sensor conductance *G*_s_ results is as follows:
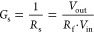


The sensor response for a *p*-type material exposed to an oxidizing gas such as NO_2_ is defined as
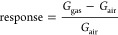
where *G*_gas_ and *G*_air_ are the steady-state conductances of the sensor in gas and in air, respectively.^43^ Commonly, when exposed to different target gases, the sensing materials can change their electronic, electrical, and optical properties. In particular, an oxidizing gas leads to an increase in conductivity for *p*-type semiconductors and a decrease for *n*-type semiconductors, vice versa for a reducing gas.^44^ The acquisition system is composed of a Keithley K2000 Multimeter to convert output voltages from analog to digital, and management/acquisition software that collects gas, temperature, and humidity sensor data. RH% control is achieved by injecting a fixed fraction of the total dry air flux into a gas bubbler filled with deionized water to create the desired humidity conditions.

As for any new sensing material, the performances of the produced Ni-functionalized bP films were probed through the so-called 3S-rule.^45,46^

Therefore, experimentation was developed through four steps to investigate the gas sensing performance of Ni/bP-based sensors.

- Selectivity. A preliminary investigation was carried out on the three 4 μm-thick samples, pristine bP, Ni/bP (**1**), and Ni/bP (**2**), exposing them to NO_2_ (1 ppm), CO_2_ (900 ppm), H_2_ (100 ppm), NH_3_ (10 ppm), CO (30 ppm), benzene (0.5 ppm), ethanol (10 ppm), ethylene (20 ppm), formaldehyde (0.2 ppm), H_2_S (10 ppm), and SO_2_ (10 ppm) diluted in dry air. Gas concentrations were chosen in order to cover different chemical species and gases relevant for environmental monitoring according to the relative TLV-TWA (threshold limit value-time weighted average).
- Sensitivity. Based on the preliminary results, the study of sensing performance of the Ni-decorated films (4 μm-thick), with different Ni NP diameters, was extended to five different concentrations of NO_2_ under dry conditions (0.1, 0.2, 0.5, 0.7, 1.0, 2.0, and 3.0 ppm). Since NO_2_ molecules tend to adsorb and react with metallic surfaces, it was necessary to employ Teflon tubing for obtaining NO_2_ concentration values in the order of tens ppb. In addition, Ni NP concentration on the bP surface was investigated for different P:Ni ratios.
- Role of humidity. To evaluate the effect of humidity on the sensing performance, Ni/bP-based sensors (4 μm-thick) were exposed to 1 ppm NO_2_ and a calibration at different RH% values was carried out. The latter was controlled inside the test chamber by a commercial Honeywell HIH-4000 humidity sensor.
- Stability. In order to investigate possible degradation of the sensing performance over time and to evaluate the influence of the film thickness on the sensing performance, Ni/bP(**1**), Ni/bP(**2**), and Ni/bP(**3**) with thicknesses of 2, 4, and 10 μm, were tested for a period of 1 month. Every week, the sensors were exposed to 1 ppm NO_2_ diluted in dry air. Dry air conditions were maintained between test runs to avoid sample contamination. In addition, the baseline resistance value in dry air has been monitored for a period of 1 month.

## Results and Discussion

### Influence of Ni on the Band Gap Energy

DFT calculations were carried out to study the influence of nickel decoration on the phosphorene band gap. First, self-consistent field (SCF) calculations were performed according to the van der Waals DFT (vdW-DF) approximation with the optB88 level for the relaxation of the structures considered. The calculated structural properties are reported in Table S1. The electronic structure of phosphorene and the band gap value were obtained by performing generalized gradient approximation with the Perdew–Burke–Ernzerhof (GGA-PBE) functional calculations. The calculated band gap value of phosphorene was 0.9 eV (see Table S2, Supporting Information), which is in agreement with a previous work.^47^

**Figure 2:**
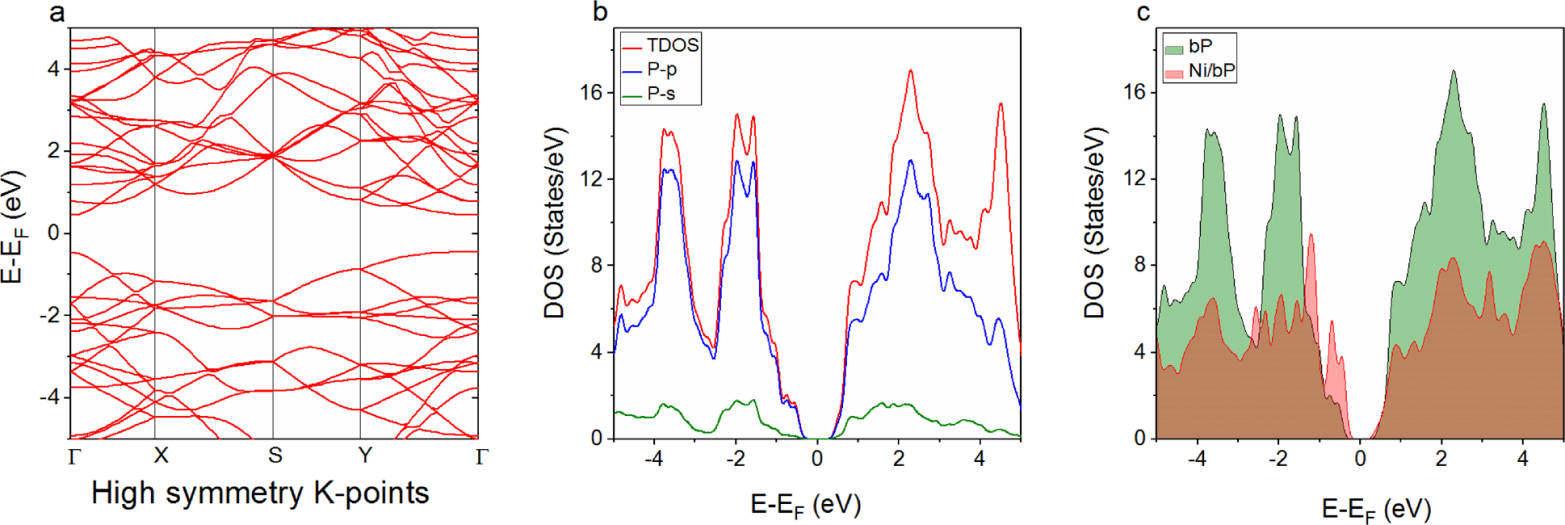
(a) Band structure and (b) TDOS and PDOS of pristine phosphorene for s and p orbitals of the phosphorus atom and (c) TDOS of pristine bP and nickel-decorated bP.

Figure 2a shows the band structure and the density of state (DOS) graphs of monolayer phosphorene. From the obtained band structure, one can observe that phosphorene possesses a direct band gap on a gamma point. Figure 2b illustrates the total and the partial density of states for s and p orbitals of phosphorus atoms. From this analysis, we can conclude that the p-character is dominant in both valence and conduction bands. By introducing Ni adatoms, the impurity state appears to be in the band gap region (Figure 2c) and the calculated band gap value is equal to 0.68 eV (see Table S2, Supporting Information). Figure 2c shows that the dominant character changed from the p-character (case of pristine phosphorene) to a hybridization of P-p and Ni-d orbitals (see Figure S3, Supporting Information). The impurity states, created in the band gap region due to the addition of Ni atoms, are located between the top of the valence band and below the Fermi level. This position allows impurity states to behave as shallow acceptor levels that can facilitate the electron exchange between the top of the valence band and the target gas.^48^ Thus, increasing hole concentration on the valence band may enhance the reactivity of the material surface.

### Synthesis of the Nanocomposites Ni/bP and Their Characterization

The liquid phase exfoliation of bulk bP was carried out in DMSO under the action of ultrasounds and afforded high quality bP flakes (see Figure S4, Supporting Information).

Two different strategies were followed for the preparation of the five nanocomposites shown in Table 1: Ni/bP (**1**), Ni/bP (**1a**), Ni/bP (**3**), and Ni/bP (**3a**) were synthesized via deposition of preformed Ni NPs on bP nanosheets, while Ni/bP (**2**) was obtained by direct growth of Ni NPs on bP.

**Table 1:** Summary of the Prepared Samples

	Ni/bP (**1**) and (**1a**)	Ni/bP (**2**)	Ni/bP (**3**) and (**3a**)
Ni NP average dimensions (nm)	11.9 ± 0.9	3.0 ± 0.8	4.5 ± 0.7
deposition method	preformed	*in situ*	preformed
Ni:bP molar ratio	1:10 (**1**) and 1:3 (**1a**)	1:10	1:10 (**3**) and 1:20 (**3a**)
film resistance [MΩ] of a fresh sample	9 (**1**) and 2.5 (**1a**)	2	7 (**3**) and 16 (**3a**)

**Figure 3:**
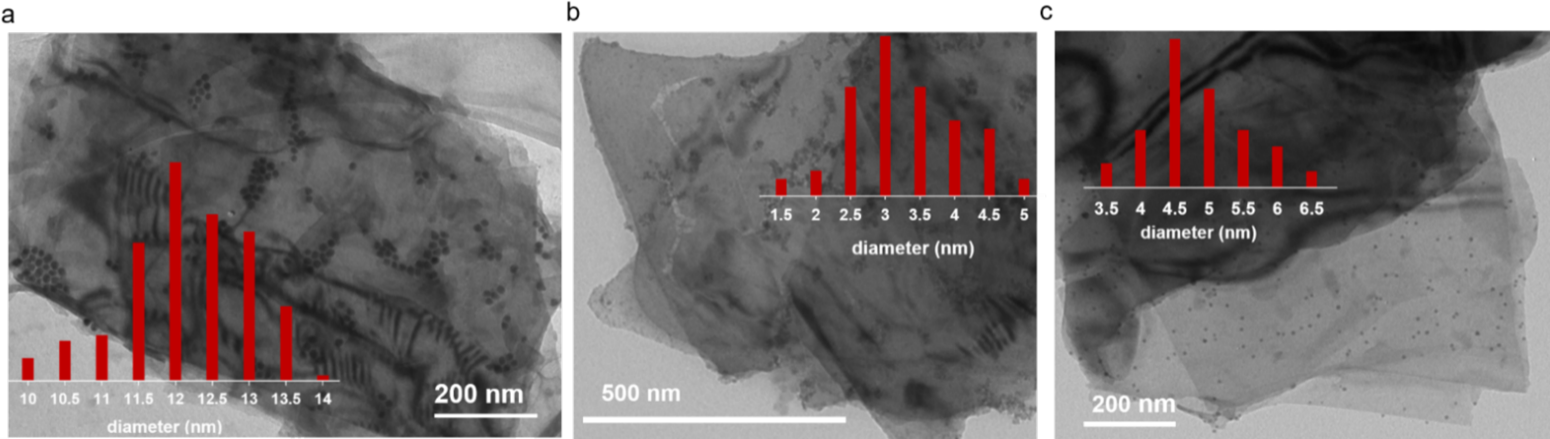
Morphological characterization of the Ni/bP samples and relative size distribution of Ni NPs. (a) TEM image of Ni/bP (**1**) on a lacey carbon copper grid, while the remaining ones are on a carbon copper grid. (b) TEM image of Ni/bP (**2**) and (c) TEM image of Ni/bP (**3**).

The morphology of the samples was studied by TEM (see Figure 3), and in all of them, the nanoparticles assume a spherical shape with a homogeneous size distribution. Looking at a large number of flakes, the physical distribution of the NPs, either grown *in situ* or preformed and then deposited, is not homogeneous on the surface, as expected, since NPs may grow or be immobilized both on the basal plane and on the edges, on the defects, and on the kinks.

Ni/bP (**1**) and Ni/bP (**1a**) (Figure 3a and Figure S5, Supporting Information) were prepared as previously described^40^ by decoration of bP nanosheets with preformed Ni NPs having an average diameter *d* = 11.9 ± 0.9 nm and using 1:10 and 1:3 molar ratios with respect to P.

In the newly prepared nanocomposite Ni/bP (**3**), preformed Ni NPs bearing a surface capping agent and with an average diameter *d* = 4.5 ± 0.7 nm were immobilized on bP nanosheets (Figure 3c). Very similar sizes were obtained by growing naked Ni NPs on bP nanosheets through mild reduction of the salt NiCl_2_·6H_2_O in the presence of NaBH_4_, thus obtaining Ni/bP (**2**), with an average Ni NP diameter *d* = 3.0 ± 0.8 nm, as shown by TEM inspection (Figure 3b). Additionally, its morphology and surface chemical composition were studied by scanning electron microscopy (SEM) (see Figure S6, Supporting Information) equipped with energy-dispersive X-ray analysis (EDAX) (see Figure S7, Supporting Information).

XRD was performed on the new nanocomposites Ni/bP (**2**) and Ni/bP (**3**), and the acquired spectra are displayed in Figures S8 and S9 in the Supporting Information, respectively. Both spectra feature the typical pattern of pristine 2D bP and show three intense peaks located at 2θ° = 16.9, 34.2, and 52.3°, corresponding to the (020), (040), and (060) reflections and confirming the retainment of the orthorhombic crystal structure of bP after functionalization.^49^

**Figure 4:**
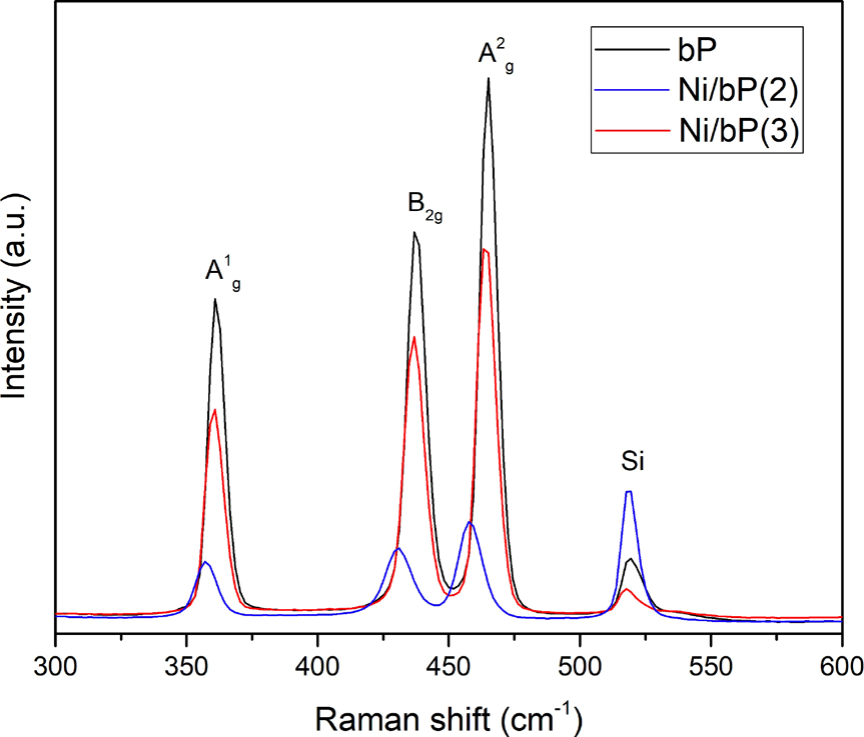
Comparison of the Raman spectra of pristine bP, Ni/bP (**2**), and Ni/bP (**3**).

Given the broad range of flake thickness, micro-Raman spectra were measured for a large number of flakes each sample (Figure 4), thus avoiding a frequency shift of the signal with varying thickness. The Raman spectrum of Ni/bP (**3**) features three characteristic peaks at 360.7, 436.7, and 464.3 cm^–1^, corresponding to the A^1^_g_, B_2g_, and A^2^_g_ phonon modes of exfoliated bP, respectively,^50^ and the pattern results are superimposable to the one of bare bP. Intriguingly, the Raman spectrum of Ni/bP (**2**) shows much broader peaks, as confirmed by the increased FWHM in comparison to bare bP and Ni/bP (**3**) (see Figure S3 in the Supporting Information), and all the three peaks are red-shifted compared to pristine bP by 4.4, 6.5, and 7.6 cm^–1^, respectively. This behavior may arise from the strong interfacial interaction between the P atoms of bP and the naked surface of Ni NPs, which partially impedes the oscillations of P atoms, reducing the vibrational energy of the P atoms coordinated to nickel with respect to the P atoms non-interacting with Ni NPs. The PXRD and Raman spectrum of Ni/bP (**1**) have been reported elsewhere.^32,25^

**Figure 5:**
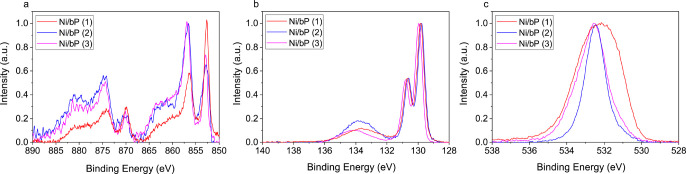
XPS spectra of core level (a) Ni 2p, (b) P 2p, and (c) O 1s on Ni/bP samples as synthetized.

The chemical state of Ni NPs in the newly prepared samples was studied by XPS, shown in Figure 5. The core level Ni 2p spectra in all the samples show a peak at a binding energy B.E. = 852.8 eV, attributed to metallic nickel, and a peak at a B.E. = 856.9 eV due to unavoidable surface oxidation of Ni NPs during manipulations of the samples.^51^

Notice that the P 2p core level XPS spectra show the spin-orbital splitting doublet with the P 2p_3/2_ peak at B.E. = 129.9 and 129.6 eV and the P 2p_1/2_ peak at B.E. = 130.7, 130.6, and 130.8 eV for Ni/bP (**1**), Ni/bP (**2**), and Ni/bP (**3**), respectively. The slightly reduced value of B.E. for Ni/bP (**2**) suggests a moderate charge transfer from Ni to bP, which is absent in Ni/bP (**1**) and Ni/bP (**3**), in agreement with the stronger interaction between P and Ni evidenced as well by Raman measurements (see Figure 4). The low-intensity peak at a B.E. = 134.1 eV is due to the incipient formation of P*_x_*O*_y_*, which becomes prevalent after 1 month of actual use of the sensor^34^ (Figure S10, Supporting Information). Core level O 1s highlights a single broad peak centered at a B.E. = 532.4 eV, which arises from different oxygen contributions. Indeed, at ∼532 eV, there is a B.E. related to oxygen of the Ni*_x_*O*_y_* lattice,^52^ which is due to the formation of thin (∼2 nm) amorphous oxidized Ni, and also to the contribution of the P-O-P lattice^53^ due to the partial oxidation of the bP surface. The broadening of the peak to higher energy is due to the presence of O 1s contribution related to strongly chemisorbed oxygen (O^–^) at the Ni NP surface and to OH^–^ groups and H_2_O absorbed on bP and Ni surfaces.^54−56^ The broadening of the peak to lower energy is probably due to P=O contribution,^53^ which is more pronounced in Ni/bP (**1**) and Ni/bP (**3**) due to the use of TOP as a capping agent.

The presence of oleylamine on the Ni NP surface of Ni/bP (**1**) and Ni/bP (**3**) is confirmed by the peak at a B.E. = 402.1 eV in the core level N 1s spectrum of the samples (see Figure S11, Supporting Information).

### Gas Sensing Performance

**Figure 6:**
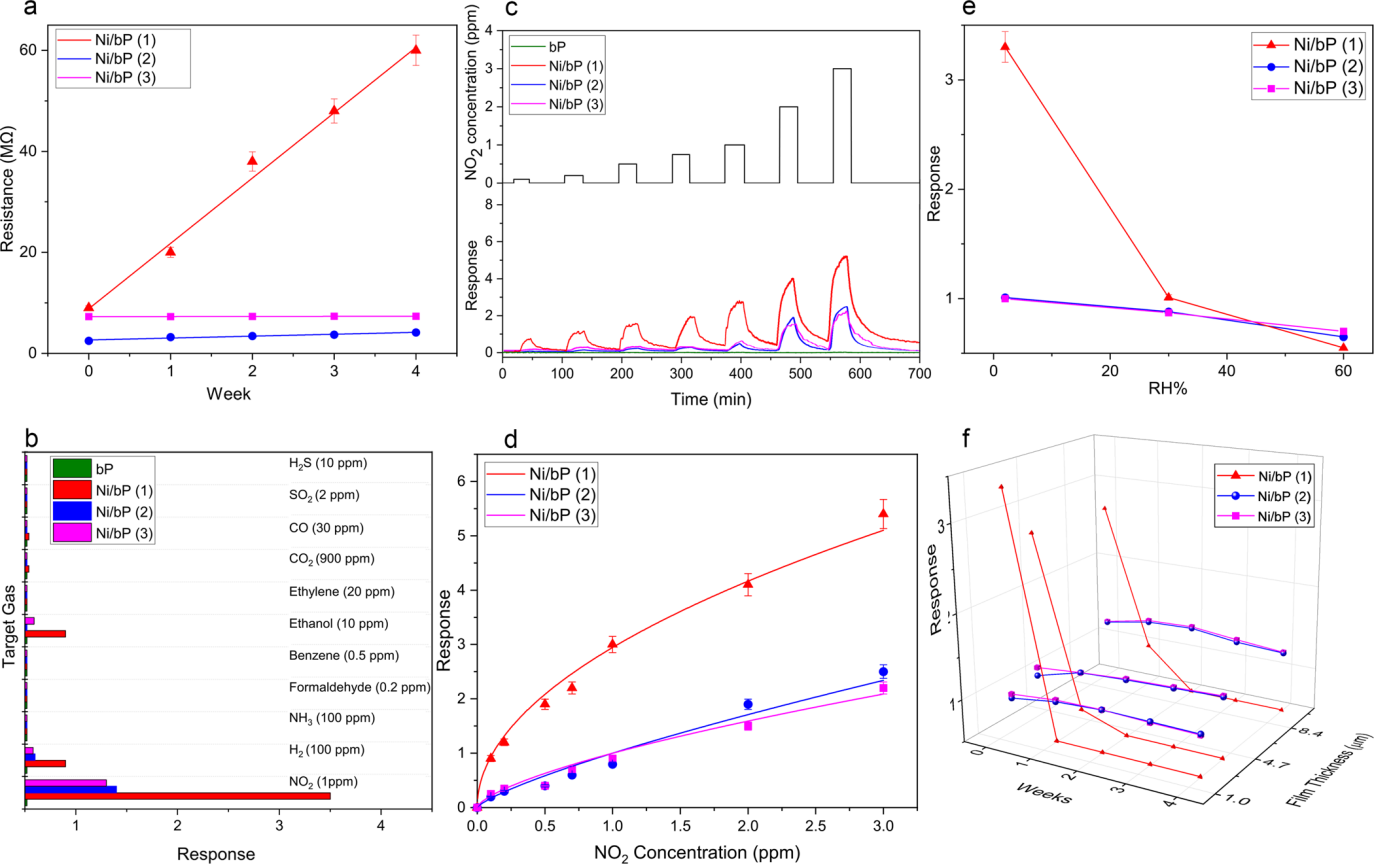
(a) Ni/bP-based films resistance in dry air for a 4 week period. (b) Selectivity of Ni/bP-based films toward different gas analytes. (c) Electrical characterization at room temperature of bP, Ni/bP (**1**), Ni/bP (**2**), and Ni/bP (**3**) devices under dry conditions with five different concentrations of NO_2_. (d) Calibration curve of Ni/bP-based sensors under dry conditions. (e) Response to 1 ppm NO_2_ at different RH% values. (f) Response values for different thicknesses of the Ni/bP-based films during a 4 week testing to 1 ppm NO_2_ under dry conditions.

To determine the effects of nickel decoration on the gas sensing properties of bP, the electrical resistance variation of the films was investigated as a function of the Ni NP dimensions, concentration, and deposition technique and compared to pristine bP. Preliminary evaluation of film resistance for the 4 μm-thick films in dry air was carried out (Figure 6a and Table 1), highlighting that the introduction of Ni nanoparticles increases carrier mobility with respect to the 4 μm-thick film of pristine bP (*R*_air_ = 18 MΩ) with a further decrease in resistance observed for all the tested samples Ni/bP (**1**), Ni/bP (**2**), and Ni/bP (**3**). This is a desirable quality for any functionalized material for gas sensing application at room temperature, already observed in similar materials such as Ni-decorated graphene,^57^ and it could be attributed to the strong interfacial interaction between metallic nickel and bP sheets. Notably, besides the concentration and dimension of Ni nanoparticles on the bP surface, the amount of TOP used during the synthesis to stabilize the colloids also affects the electrical resistance of Ni/bP samples. Indeed, the capping agent TOP on samples Ni/bP (**1**) and Ni/bP (**3**) works as a dielectric layer that impacts negatively on the resistance of the material. However, the baseline drift variation in Figure 6a highlights that Ni/bP (**1**), with an average diameter of NPs of 12 nm, is electrically unstable after 1 week of use. In contrast, Ni/bP (**2**) and Ni/bP (**3**) devices show better stability than Ni/bP (**1**), and Ni/bP (**3**) further highlights a lower baseline drift in dry air due to the protective role of TOP. Measurements of pristine bP baseline variation was impossible to monitor during the 4 week period due to the fast degradation of the material under dry air conditions that affects dramatically the electrical properties of the sensing film.

### Selectivity

To prove the potential effect of the decoration technique (immobilized or grown *in situ*) on the selectivity of Ni/bP films, devices produced with Ni/bP (**1**) and Ni/bP (**2**) samples were exposed to several gases and vapors of liquids in air at room temperature, i.e., formaldehyde, ammonia, ethanol, ethylene, benzene, nitrogen dioxide, carbon monoxide, carbon dioxide, sulfur dioxide, hydrogen, and hydrogen sulfide (Figure 6b). Among the gases tested, the Ni/bP nanocomposites demonstrated a high selectivity only toward NO_2_ with an experimental LOD of 100 ppb, whereas the pristine bP sensor exhibited a negligible response to all tested gases in dry air and, for this reason, it is not shown in Figure 6b.

Meanwhile, the Ni deposition technique was not significantly impacting on the selectivity of the film, and the dimension of Ni NPs resulted as the key parameter governing the response intensity as explained hereafter.

### Sensitivity

From the measurement of the dynamic response of Ni/bP (**1**), Ni/bP (**2**), Ni/bP (**3**), and pristine bP exposed to various concentrations of NO_2_, Ni/bP (**1**), having bigger NPs, shows an increased sensor response (see Figure 6c).

Samples Ni/bP (**2**) and Ni/bP (**3**), having Ni NPs with the same average dimensions (see Table 1), concentration, and film thickness, show similar sensitivity regardless of the deposition technique used.

As previously reported, pristine bP can exhibit a sensitive response to NO_2_ and it has been theoretically predicted and experimentally observed that the adsorption of NO_2_ molecules on the bP surface can take place via noncovalent interactions.^58^

Our study proves that functionalization with Ni NPs induces significant chemical sensitization of the bP surface toward the sensing of NO_2_ and satisfyingly nickel-decorated bP exhibited a highly stable baseline resistance with a negligible noise level and a selective response to NO_2_ with a positive conductance variation. The baseline noise level is drastically improved with Ni/bP (**2**) (∼0.002%) compared to pristine bP (∼0.25%), Ni/bP (**1**) (∼0.4%), and Ni/bP (**3**) (∼0.2%) (see Figure S12, Supporting Information) since the residual presence of oleylamine and TOP in the latter samples affects the electrical conductance of the film. This result suggests that the presence of metallic channels can provide an intrinsically lower noise compared to semiconducting channels, inducing a large signal variation with a low baseline resistance noise.

The response (τ_res_) and recovery times (τ_rec_) of Ni/bP films, calculated as the times necessary to attain 90% of the steady-state sensor response and as the e-folding response, respectively, ranged from 25 to 40 min (Figure 6c). Thus, the characteristic times of Ni/bP-based sensors are comparable to those of other bP-based chemiresistive gas sensors (Table 2), even if τ_res_ and τ_rec_ are influenced by the experimental setup, including gas carrier nature, such as N_2_ or synthetic air. The slow and incomplete recovery of the baseline shown in Figure 6c, especially for Ni/bP (**1**), was previously observed in few-layer reduced graphene oxide^59^ and in few-layer black phosphorus-based sensors.^14,60^ This was explained by the presence of irreversible sites where target gas molecules may strongly bind, physically represented by defects, inhibiting a complete desorption in the time scale of a sensing cycle.^14,61^

**Table 2:** Comparison of Recently Reported bP-Based NO_2_ Gas Sensors

material	sensing response^a^	τ_res_ (min)	τ_rec_ (min)	atmosphere	ref
bP	50% (1 ppm)	∼60	∼60	N_2_	(14)
bP	88% (100 ppb)	∼5	∼6	N_2_	(62)
Ni/bP (**1**)	100% (100 ppb)	∼25	∼40	air	this work
Ni/bP (**2**)	30% (100 ppb)	∼25	∼40	air	this work
Ni/bP (**3**)	30% (100 ppb)	∼25	∼40	air	this work
Pt/bP	90% (100 ppm)	∼5	∼10	N_2_	(25)
ZnO/bP	74% (50 ppb)	∼11	∼10	air	(17)

a All sensing responses were normalized as follows: sensing response (%) = |(*X*_gas_ – *X*_carrier_)/*X*_carrier_| × 100%, where *X*_gas_ and *X*_carrier_ are the values of the selected quantity when the sensor is exposed to an analyte and a carrier gas, respectively.

The calibration curves in Figure 6d that display the Ni/bP (**1**), Ni/bP (**2**), and Ni/bP (**3**) responses vs NO_2_ gas concentrations show typical fit with Langmuir isotherms for molecules adsorbed on a surface,^58^ which suggests that charge transfer is the leading mechanism for NO_2_ sensing in multilayer bP-based devices.

In the same gas environment, the load of Ni on bP could affect, either positively or negatively, the response of the composites to NO_2_. In fact, enhancing the Ni NP concentration at the surface, i.e., going from a P:Ni ratio = 10 to a P:Ni ratio = 3, produces a decrease in the sensor response. This trend can be ascribed to the partial aggregation of Ni nanoparticles that led to a reduction of the active surface with a subsequent decrease in active sites. On the other hand, Ni/bP (**3a**) with a P:Ni ratio = 20 gives a reduced response, 10 times less, compared to Ni/bP (**3**) having a P:Ni ratio = 10 due to the lower concentration of Ni NPs and the consequent scarce availability of Ni active sites (see Figure S13, Supporting Information).

### Role of Humidity

To investigate the influence of humidity on the response, the homebuilt sensors were exposed to 1 ppm NO_2_ in the presence of different RH% conditions, from 2% up to 60% (Figure 6e). Ni/bP (**2**)- and Ni/bP (**3**)-based samples showed a small decrease in the response value with increasing RH%, highlighting good humidity resistance. On the contrary, the Ni/bP (**1**) film showed a rapid decline in sensing performance and an irreversible degradation of the film at 30% RH. In the case of 60% RH, twin devices were used and irreversibly compromised after the water vapor injection.

### Stability

To demonstrate the ambient stability of these sensors and to investigate the influence of the film thickness on the sensing performance, three sensors (2, 4, and 10 μm thick), based on each Ni-decorated bP film, were produced and tested with 1 ppm NO_2_ for a 4 week period under dry conditions (Figure 6f). Interestingly, despite the initial higher response, Ni/bP (**1**) already suffered a decreased performance after 1 week under a dry air flux owing to the fast oxidation process that degrades the material. XPS on the exposed film highlighted that most P and Ni was completely transformed into the corresponding oxides (Figure S11, Supporting Information). This behavior is slightly impeded and slowed down in thicker films, as shown in Figure 6f.

Satisfyingly, Ni/bP (**2**) and Ni/bP (**3**) show better stability over the measurement period, even for thinner films. A possible explanation of the described behavior lies in the morphological characteristics of the employed materials. In fact, the nonhomogeneous decoration of Ni NPs on the surface of bP can impact on the stability of the sensing film, especially with different Ni NP diameters. In addition, Ni NPs in Ni/bP (**1**) have an average diameter of ∼12 nm, which increases the interlayer separation between bP flakes with respect to Ni/bP (**2**) having NPs with a diameter that is four times smaller. This fact drastically enhances the specific surface area exposed to NO_2_ in the case of Ni/bP (**1**), resulting in a more effective initial sensor response. On the other hand, this separation exposes as well a more extended sensing area to ambient adsorbates,^63^ including water molecules in the case of RH% experiments, which irreversibly react with bP forming oxidized phosphorus species (P*_x_*O*_y_*), altering the electronic properties of the material. Interestingly, the samples Ni/bP (**2**) and Ni/bP (**3**) show a stable response for a 4 week measurement period with the response having no significant dependence on the film thickness. Ni decoration effectively suppresses ambient degradation for at least 1 week in Ni/bP (**1**) and over 4 weeks in Ni/bP (**2**) and Ni/bP (**3**). This result highlights that, at equal Ni concentrations, the NP dimensions play a crucial role in the stability of the bP-based gas sensor, and thus Ni/bP (**2**)- and Ni/bP (**3**)-based devices can be practically used under ambient conditions for a reasonable period without a considerable performance degradation.

### Proposed Sensing Mechanism

After decorating the bP surface with nickel, the sensor response to NO_2_ gas molecules is markedly enhanced with respect to the pristine bP-based sensor and the involved gas sensing mechanism is supposedly different. Ni NPs play a dominant role in the adsorption/desorption equilibrium of O_2_ and NO_2_ behaving as receptors for NO_2_ molecules, while bP transduces this interaction in an electrical conductance variation at RT. In detail, the adsorption of NO_2_, which is an oxidizing gas, on Ni NPs leads to the formation of NO_2_^–^. In this process, NO_2_ molecules can exchange electrons directly with the shallow acceptor levels ionized by the top of the valence band (see the DFT study). This electron transfer leads to an increase in hole density that finally results in an increase in conductance.

Oxygen molecules coming from the atmosphere are chemisorbed on the Ni surface. After, the chemisorbed O_2_ molecules can react with electrons at the surface and transform them into activated oxygen ions (O^–^), which is the predominant species formed in the adsorption at RT,^64−66^ thus decreasing the electron density and forming a depletion layer, according to the following equation:
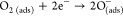
1

When NO_2_ is injected, being a strong oxidizing gas and having a stronger electron affinity than activated oxygen, it attracts electrons from the surface and it is chemisorbed according to the following equation:
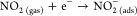
2thus creating an increase in hole concentration on the surface of Ni NPs. The fast hole transfer from the thin (∼2 nm) amorphous Ni*_x_*O*_y_* layer formed at the NP surface (−4.64 eV of the valence band level)^67^ to the bP (−4.40 eV of the work function level)^68^ will enhance the response time and sensitivity toward NO_2_. This behavior has been observed in similar systems involving reduced graphene oxide.^69^

**Figure 7:**
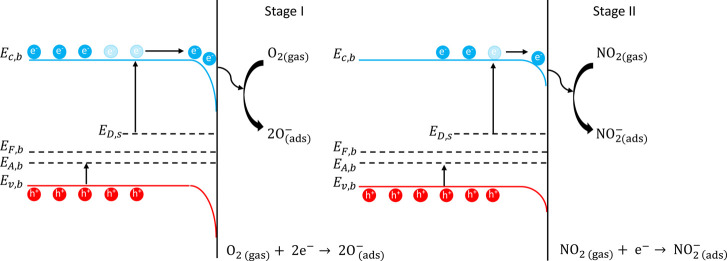
Energy band diagram illustrating the sensing mechanism of Ni/bP toward NO_2_. *E*_c,b_ and *E*_v,b_ are the bulk conduction and valence bands and *E*_F,b_, *E*_A,b_, and *E*_D,s_ are the bulk Fermi, bulk shallow acceptor, and surface donor levels, respectively.

The above-discussed adsorption process can be confirmed and schematized by the energy band diagram reported in Figure 7, which shows the calculated band structure before and during the analyte gas interaction. During the adsorption of oxygen from the surrounding atmosphere (stage I), the electrons accumulate at the surface of the material, creating a depletion layer (band bending effect) impacting on the electron affinity, reaching an equilibrium represented by the sensor baseline.^70^ When the analyte is introduced (stage II), NO_2_ molecules interact with the electrons at the surface, decreasing the potential barrier created during the first stage, resulting in an increase in the conductivity of the material with a subsequent change in the band bending that is transduced in a sensor response.

The desorption process can take place either by surface desorption, related just to the charge transfer and consequently to the binding energies reported by Kou et al.,^8^ and/or by degassing NO_2_ molecules from the nanocomposite layers. It is reasonable to assume that the fastest process involves surface desorption, while NO_2_ diffusion phenomena between bP layers occur with a slower rate.^14^ When the sensing film is exposed to the gas, NO_2_ molecules tend to diffuse between the material layers and the interlayer desorption results in a slower process than the one from the exposed Ni/bP surface, as shown for pristine bP and in other 2D systems like graphene.^61^ Therefore, we attribute the enhanced sensing properties of the Ni/bP nanocomposites with respect to the pristine material to the synergism between bP and Ni NPs. In our case of study, the deposition of Ni NPs on bP plays a crucial role as an active center in the charge carrier transport to adsorb NO_2_ molecules with strong electron-withdrawing power. The electron transfer from bP via Ni nanoparticles to adsorbed NO_2_ molecules produces a hole enrichment in a *p*-type hole-transport-driven composite and consequently increases its conductance. Meanwhile, bP allows room-temperature operation acting as a transducer for the electric signal.

## Conclusions

In summary, the functionalization of bP nanosheets with Ni NPs was carried out following two different strategies. We could prove that robust and electrically active Ni/bP-based films can be prepared while the structural properties of the pristine phase are preserved. The possibility of spin-coating these films, based on the selection of the best thickness, represents an important technological advantage with respect to other deposition techniques owing to the large-scale production and affordability of this method.

For the first time, we experimentally demonstrated the gas sensing properties of the fabricated Ni/bP films and the resulting devices showed a chemiresistive behavior, exhibiting the properties of high sensitivity and selectivity toward NO_2_, reaching a 100 ppb experimental LOD with full reversibility of the reaction. These characteristics in conjunction with the technological advantage to operate at room temperature make Ni/bP competitive with respect to other materials currently in use, such as the well-known metal oxide-based sensors that require high activation energy.

It was shown that the decoration method (preformed or *in situ*) applied to pristine bP marginally affects the electrical properties of the sensing films. Comparing the samples with the same Ni NP dimensions, the capping agent TOP acts as a dielectric layer, increasing the material resistance and the baseline noise level. In addition, we proved the influence of Ni NP concentration, highlighting that a P:Ni ratio of 10:1 was the most effective to achieve a reliable sensing performance.

We also proposed a model to rationalize the conductance variation measured during the NO_2_ calibration of Ni/bP sensors in terms of hole-transport-driven conduction. This sensing mechanism finds analogy with the behavior of chemiresistive gas sensors based on *p*-type semiconductors, in which the charge transport is ascribed to positive carriers.

In conclusion, the almost unaltered performance of the Ni/bP (**2**) and Ni/bP (**3**) samples under ambient conditions for a month, contrary to Ni/bP (**1**), highlighted the crucial role of Ni NP dimension in film stability. This feature was not obvious for bP-based devices, the latter being subjected to easy ambient degradation, especially under wet conditions. However, the produced devices show quite long response/recovery times. Therefore, a combination of an effective bP nanosheets deposition togheter with the control of the size distribution of Ni NPs can be further optimized to aid the adsorption/desorption kinetics, improving the performance of the final device.^60^

In conclusion, we believe that this work can open the way toward real applications of Ni/bP films in high performance and wearable sensors, which represent the next generation of electronics technology.^71^

## Supporting Information Available

Additional experimental details, material characterization, and methods, including schematization of experimental setup, computational details of performed DFT simulations, TEM, SEM, XRD, XPS, and electrical characterization additional data (PDF)
