# Supplementary material for: Air Stable Nickel-Decorated Black Phosphorus and Its Room-Temperature Chemiresistive Gas Sensor Capabilities
Source: ACS Appl Mater Interfaces. 2021 Sep 10;13(37):44711–22. doi: 10.1021/acsami.1c10763 (PMC8461602; doi:10.1021/acsami.1c10763)
Supplement: Supplementary file 1 — am1c10763_si_001.pdf [file am1c10763_si_001.pdf]

Supporting Information

Air stable nickel-decorated black phosphorus and its room-temperature chemiresistive gas sensor capabilities

Matteo Valt[1*], Maria Caporali[2*], Barbara Fabbri[1], Andrea Gaiardo[3], Soufiane Krik[1,3], Erica Iacob[3], Lia Vanzetti[3], Cesare Malagù[1], Martina Banchelli[4], Cristiano D'Andrea[4], Manuel Serrano-Ruiz[2], Matteo Vanni[2], Maurizio Peruzzini[2] and Vincenzo Guidi[1]

1 Department of Physics and Earth Sciences, University of Ferrara, Via G. Saragat 1/C, 44122, Ferrara, Italy.

2 Istituto di Chimica dei Composti Organometallici, Consiglio Nazionale delle Ricerche (CNR ICCOM), Via Madonna del Piano 10, 50019 Sesto Fiorentino, Italy.

3 MNF - Micro Nano Facility unit, Sensors and Devices center, Bruno Kessler Foundation, Via Sommarive 18, 38123, Trento, Italy.

4 Istituto di Fisica Applicata "Nello Carrara", Consiglio Nazionale delle Ricerche (CNR IFAC), Via Madonna del Piano 10, 50019 Sesto Fiorentino, Italy.

* Corresponding Authors e-mail: matteo.valt@unife.it; maria.caporali@iccom.cnr.it

GAS MEASUREMENTS SETUP

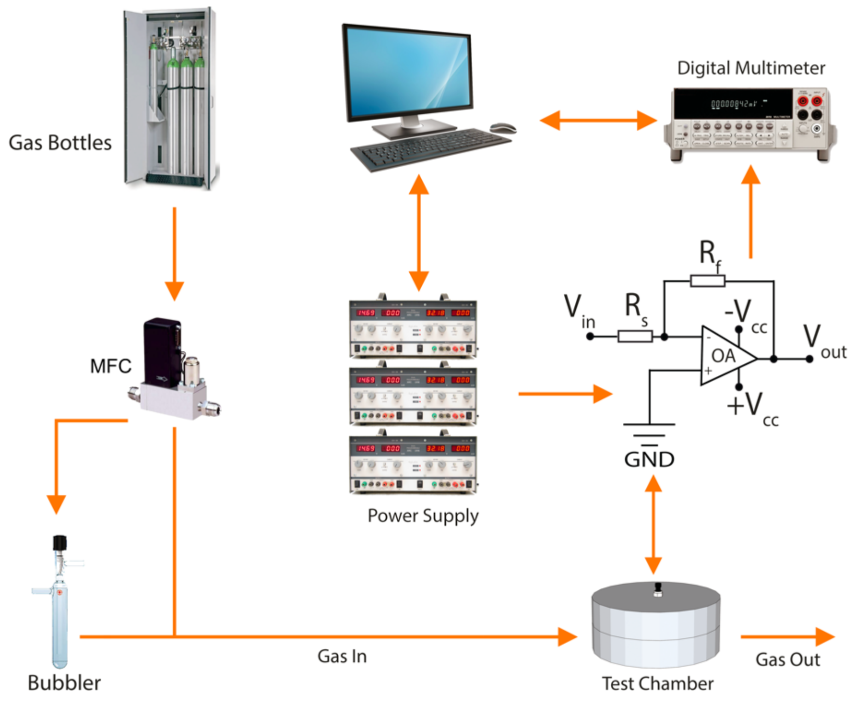

Figure S1. Schematic representation of the gas mixing system composed by certified gas bottles, mass flow controller (MFC), bubbler for humidity control and sealed gas measurements chamber. Data acquisition system composed by suitable electronics, digital multimeter and power supply.

COMPUTATIONAL DETAILS

In Figure S2 it is shown the model structure used in this work for a phosphorene monolayer. Bulk black phosphorus belongs to the space group Cmce (No. 64) with a = 3.3164 Å b = 10.484 Å c = 4.3793 Å.[1] The self-consistent field (SCF) cycles were performed using 37 and 333 Ry for the cut-off energies for the wave functions and charge density, respectively. The sampling of the Brillouin zone was carried out according to the Monkhorst-Pack scheme and the number of k points was optimized to obtain a good convergence in the calculations and the optimized combination was set to 6*1*4. To study the impact of Ni atoms on the band gap of a phosphorene monolayer, a (2*1*2) supercell was generated, which gave us a structure containing in total 16 P atoms. Then, we placed the Ni atom in the hollow (H) site at 1.01 Å as a vertical height of the adatom from the phosphorene layer. We have chosen the H site because it is the most stable adsorption site for Ni, and the distance between the Ni adatom and the phosphorene layer has been chosen according to the study done by T. Hu et al.[2] All the structures used in the calculations were relaxed by minimizing the forces acting on each atom reaching values below 1 mRy/a.u. In order to treat the van der Waals interaction, the vdW-DF approximation with the optB88 level was adopted during the relaxation process. The convergence criteria have been fixed for all calculations in this section, and it is set to 10-6 Ry for the total energy of the system. After the relaxation process, we have optimized the structural properties of phosphorene. The obtained results are in good agreement with the literature, both with experimental and simulated results.[1,3] Table S1 shows the structural parameters simulated in this work and those found in literature for monolayer phosphorene.

$$\theta_1 = (p_2\widehat{p_3p_4});\ \theta_2 = (p_1\widehat{p_2p_3});\ L_1 = p_5 - p_6;\ L_2 = p_2 - p_3$$

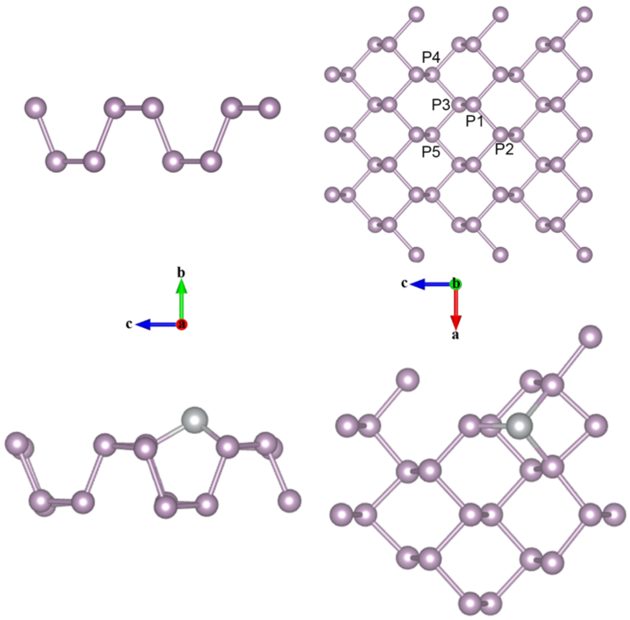

Figure S2. Side and top view of phosphorene (top) and nickel decorated phosphorene (bottom). $\theta_1 = (p_1\widehat{p_3p_5});\ \theta_2 = (p_4\widehat{p_3p_5});\ d_1 = p_1 - p_2;\ d_2 = p_1 - p_3$

Table S1. Comparison of structural properties of phosphorene calculated in this work and those reported in literature. *data converted to Cmne for better comparison*

		This work	Literature	
			Calc. [1]	Exp. [4] *
Lattice parameters	*a* (Å)	3.310	3.298	3.316
	c (Å)	4.57	4.627	4.38
Bond angle & length	θ_1 (°)	104.07	104.1	102.11
	θ_2 (°)	95.85	95.9	96.38
	d_1 (Å)	2.23	2.22	2.22
	d_2 (Å)	2.26	2.26	2.24

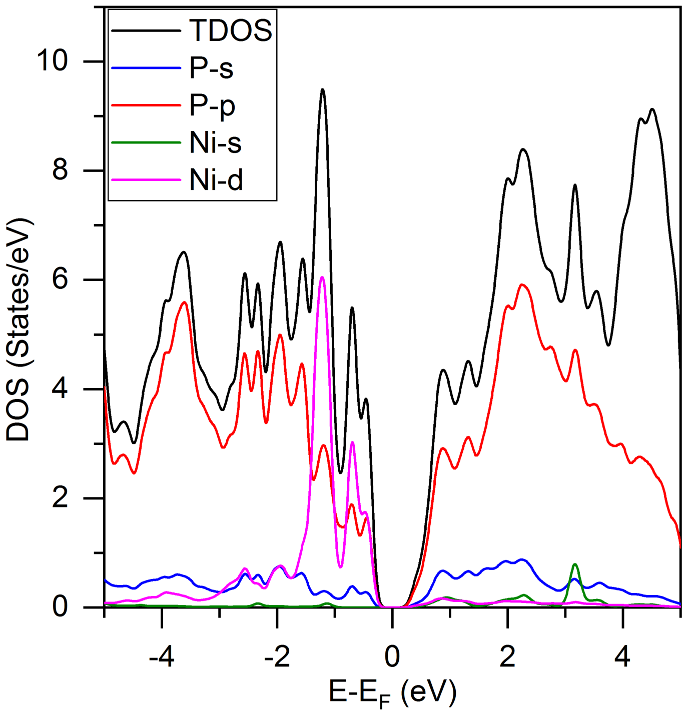

Figure S3. Total and partial density of state of Ni decorated bP.

Table S2. The calculated band gap using GGA-PBE approximation for both phosphorene and Ni decorated phosphorene.

	Phosphorene	Ni/Phosphorene
Eg (eV)	0.9	0.68

MATERIAL CHARACTERIZATION

Transmission electron microscopy. TEM studies were carried out using a Philips instrument operating at an accelerating voltage of 100 kV. Few drops of pristine bP, Ni/bP (**1**), Ni/bP (**1a**) and Ni/bP (**2**) suspension in tetrahydrofuran were placed on the TEM copper/carbon grid, air dried, and measured.

Scanning electron microscopy. SEM and STEM experiments were carried out using a Dual Beam, TESCAN GAIA3 FIB/SEM ultrahigh resolution field emission microscope. Few drops of Ni/bP (**2**) suspended in tetrahydrofuran were placed on the TEM copper/carbon grid, air dried, and measured. The instrument is equipped with an EDS X-ray microanalysis system (EDAX, AMETEK, Mahwah NJ, USA, software TEAM EDS Basic Software Suite) that allowed the chemical analysis of Ni/bP (**2**).

Imaging of the sample Ni/bP (**3**) was performed by using the same instrument in STEM mode, dropcasting few drops of Ni/bP (**3**) suspension in tetrahydrofuran on the TEM copper/carbon grid.

Inductively coupled plasma mass spectrometry. ICP-MS measurements were performed with an Agilent 7700 Series spectrometer. Samples followed a microwave-assisted digestion in Nitric acid for trace analysis. Then, different dilutions of each sample with water for trace analysis were prepared, in order to obtain concentrations in the sensitivity range of the instrument for the elements under investigation (namely Ni and P). Standards at different concentrations have also been prepared and measured contextually to sample measurements, in order to obtain a calibration curve for each element under investigation.

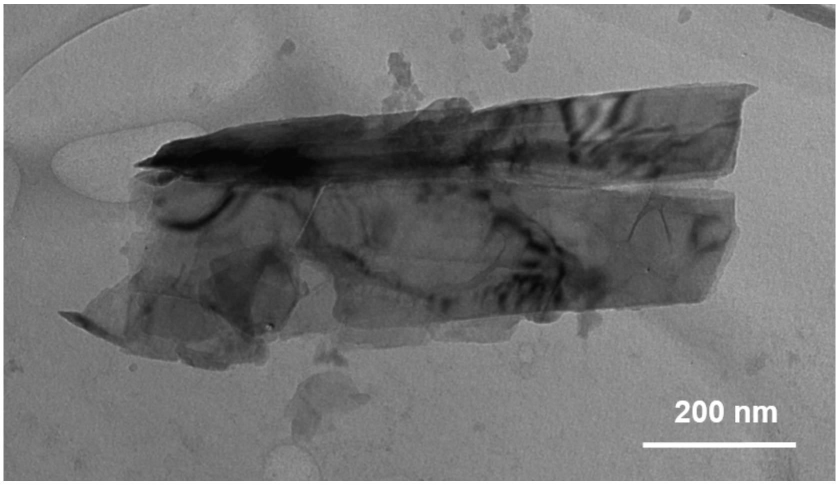

Figure S4. Bright field TEM image of few-layer black phosphorus on carbon copper grid. Scale bar = 200 nm.

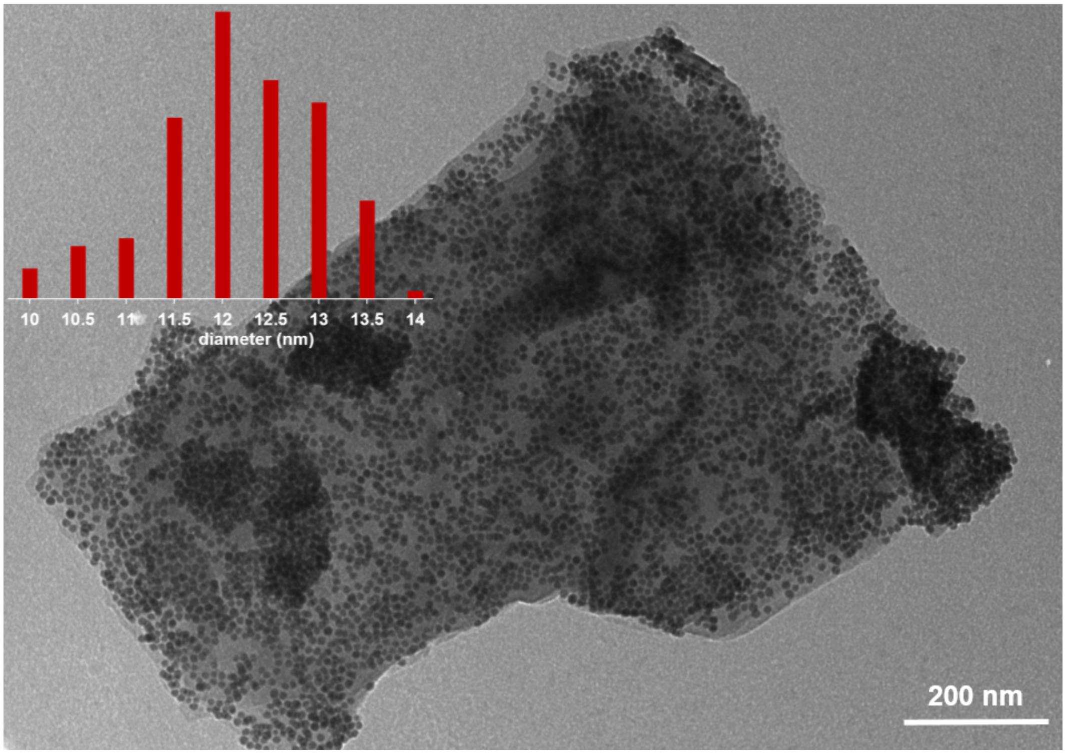

Figure S5. TEM image of Ni/bP (**1a**).

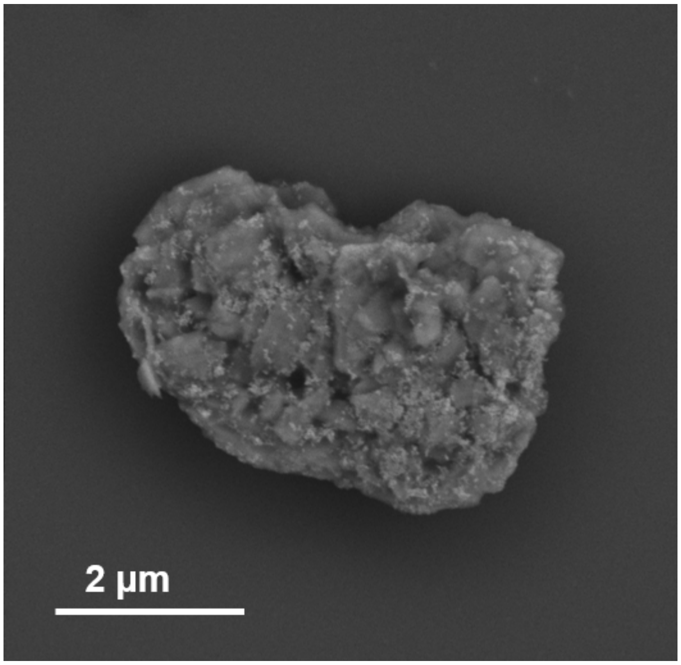

Figure S6. SEM image of bP nanosheets decorated with nickel nanoparticles grown on its surface, sample Ni/bP (**2**). Scale bar = 2 µm.

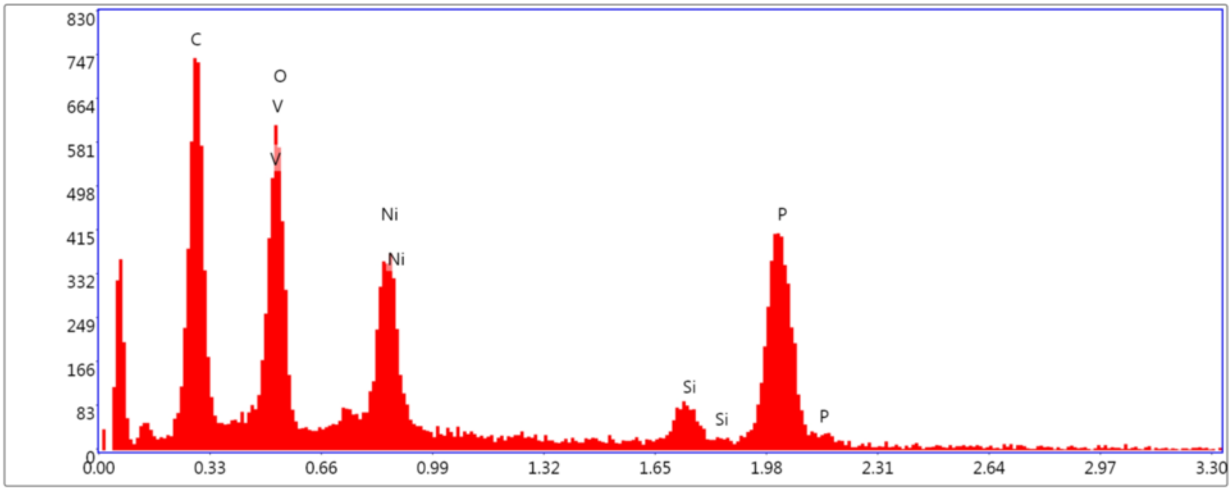

Element	Weight %	Atomic %	Net Int.	Error %	Kratio	Z	A	F
C K	28.14	48.76	80.59	11.90	0.1468	1.1895	0.4384	1.0000
V L	2.43	0.99	2.04	76.16	0.0164	0.7854	0.8624	1.0000
O K	11.36	14.77	59.95	10.29	0.0881	1.1123	0.6976	1.0000
NiL	11.92	4.23	29.76	8.65	0.0955	0.7945	1.0086	1.0000
SiK	3.49	2.58	9.78	16.10	0.0334	0.9665	0.9801	1.0113
P K	42.67	28.67	72.92	8.98	0.3887	0.9223	0.9865	1.0010

Figure S7. EDAX analysis of Ni/bP (**2**).

Powder X-ray diffraction (XRD). Data were collected with an X'Pert PRO diffractometer, operating in Bragg-Brentano parafocusing geometry with Cu-Kα radiation (λ = 1.5418) at 40 kV and 30 mA. Samples were prepared by slow drop-cast of the material suspended in acetone, directing a nitrogen stream onto the sample holder to speed up solvent evaporation. The process was continued until a uniform film of the material had formed. Data acquisition was carried out under air exposure.

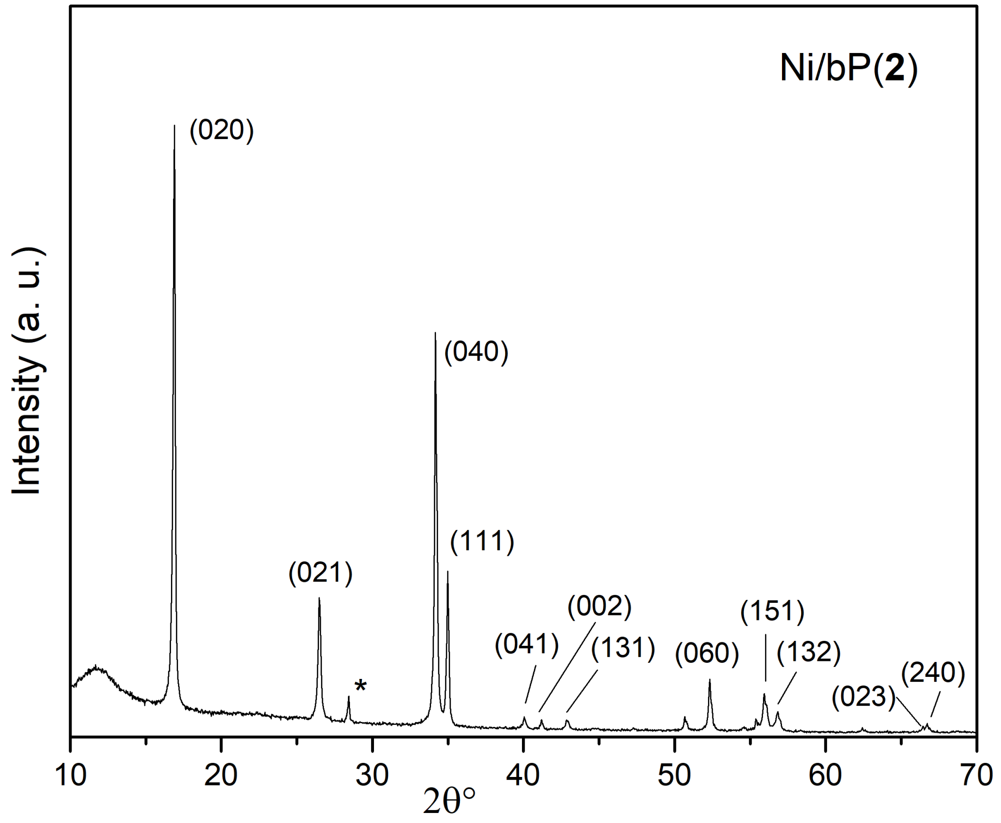

Figure S8. X-ray powder diffraction of Ni/bP(2). The peak marked with an asterisk is due to the sample-holder.

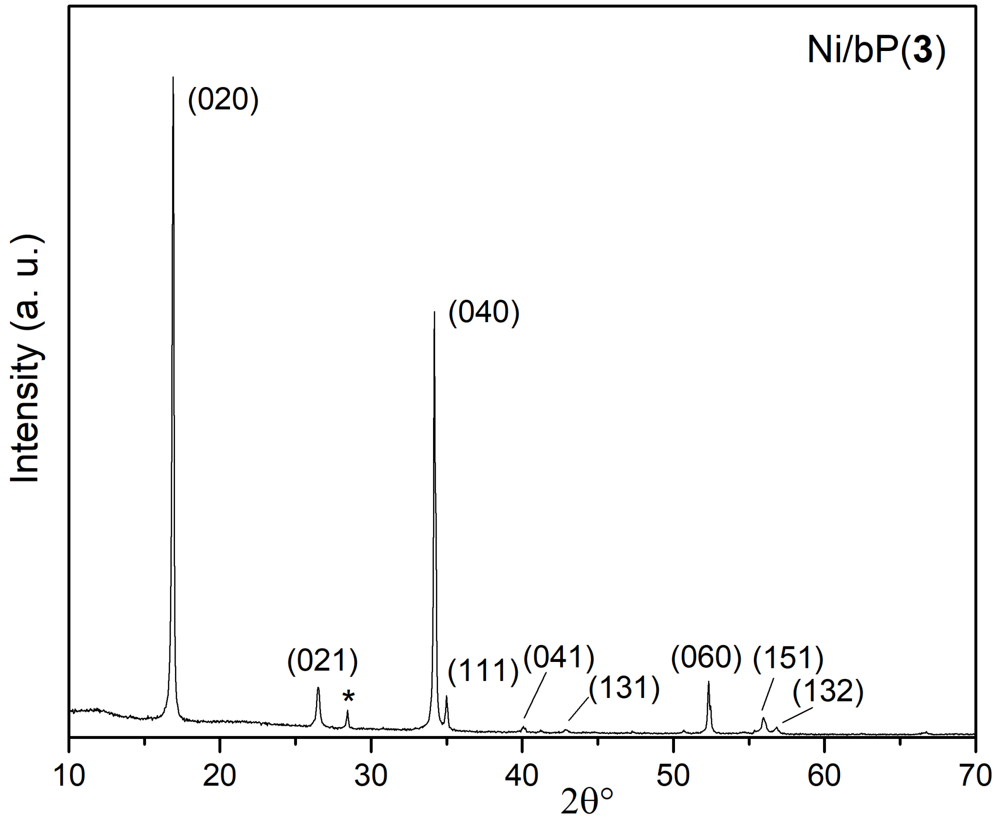

Figure S9. X-ray powder diffraction of Ni/bP(3). The peak marked with an asterisk is due to the sample-holder.

Raman scattering. Raman measurements were carried out using a micro-Horiba Xplora system coupled to a 532 nm wavelength laser. The backscattered light was collected by a 100× microscope objective with 0.9 NA, which generates a ~1-μm large laser beam waist. Integration times of 10 s, laser power values in the 1-2 mW range and a grating of 1200 cm^{-1} were employed. A suspension of 2D bP, Ni/bP(**2**) or Ni/bP(**3**) in tetrahydrofuran was dropcasted on a Si/SiO_2 wafer. After one minute of exposure, the wafers were rinsed with acetone and dried under a stream of nitrogen for 15 minutes.

Table S3. Average Raman shift and full-width at half maximum (FWHM) for bP, Ni/bP(**2**) and Ni/bP(**3**).

phonon mode	bP	Ni/bP(**2**)	Ni/bP(**3**)
A^1_g	361.5; 7.2	357.1; 11.4	360.7; 8.5
B_{2g}	437.6; 7.7	431.1; 15.1	436.7; 8.3
A^2_g	465.2; 6.9	457.6; 11.3	464.3; 7.3

Per each sample it is shown the Raman shift (cm-1) of the phonon mode (left side) and the corresponding FWHM of the signal (right side). A series of flakes (15) have been measured per each sample and the average spectrum has been obtained. The FWHM was obtained as result of the fitting with a Lorentzian function.

X-ray photoelectron spectroscopy. XPS measurements were performed using a Kratos AXIS UltraDLD instrument (Kratos Analytical, Manchester, UK) equipped with a hemispherical analyser and a monochromatic Al Kα (1486.6 eV) X-ray source, in spectroscopy mode. The phosphorene samples were analysed with a take-off angle between the analyser axis and the normal to the sample surface of 0°, corresponding to a sampling depth of approximately 10 nm. Initially a survey (in the 1300, -5 eV energy range) was recorded to identify the elements present on the surface and subsequently Ni 2p, O 1s, N 1s, C 1s and P 2p core levels were acquired with higher energy resolution. The spectra were aligned setting C 1s core level hydrocarbon peak at 285 eV. All XPS data were analyzed using the software described in Speranza and Canteri.[5]

Table S4. Elemental composition as derived from XPS shown in Figure 5 and S10.

Sample	Ni (%)	O (%)	P (%)	N(%)
Ni/bP (1)	4.3	28.0	30.0	2.4
Ni/bP (2)	0.9	23.9	6.5	0
Ni/bP (3)	0.6	25.7	16	0.5

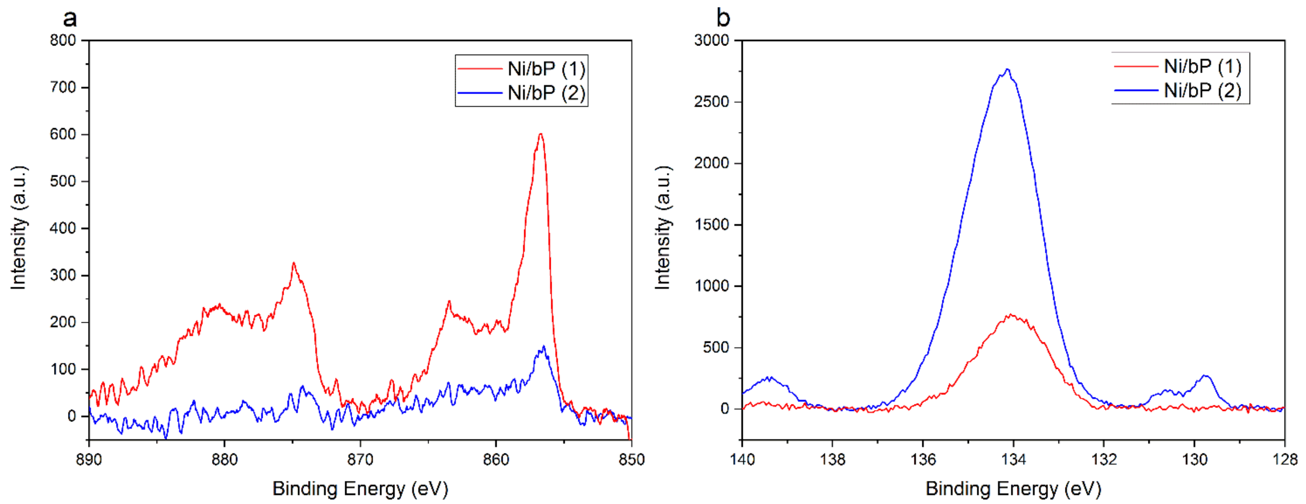

Figure S10. (a) Ni 2*p* spectra on materials used as gas sensor in operating conditions for 1 month. (b) P 2*p* spectra on materials used as gas sensor in operating conditions for 1 month.

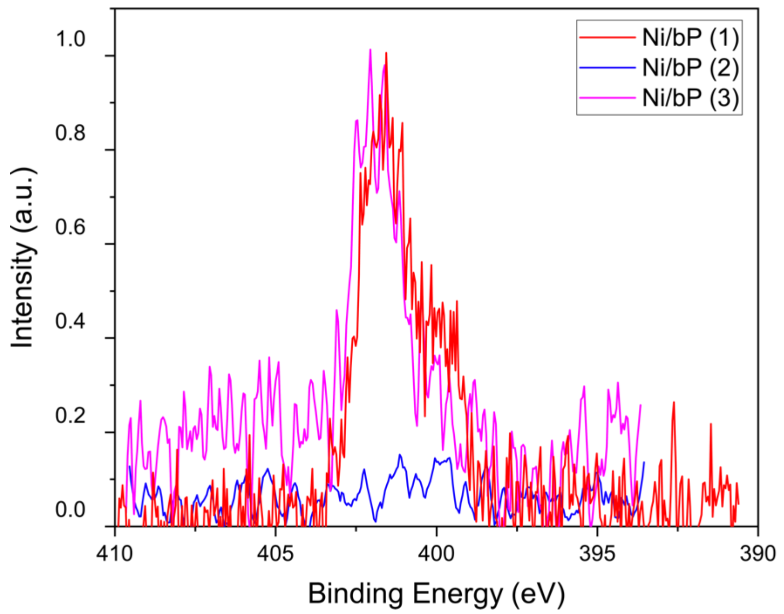

Figure S11. Nitrogen 1*s* spectra on materials as synthetized.

ELECTRICAL CHARACTERIZATION

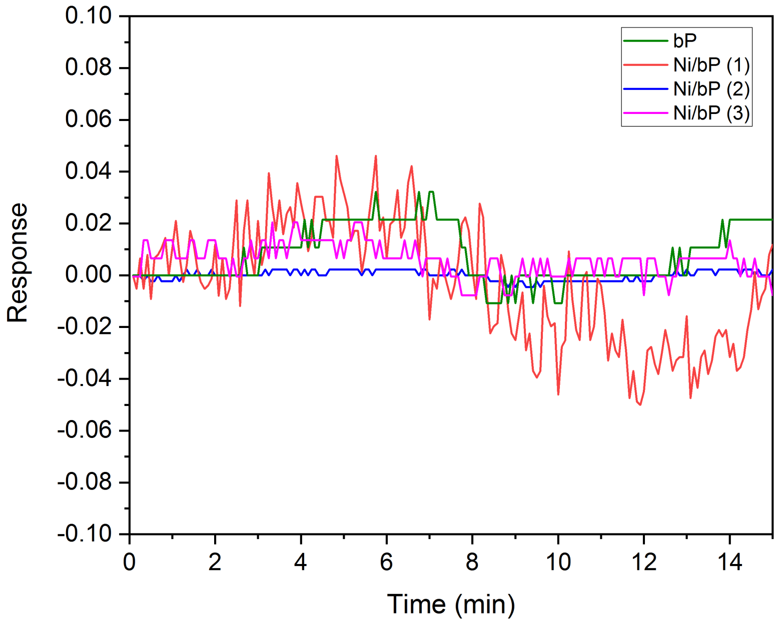

Figure S12. Baseline noise level of bP, Ni/bP (**1**), Ni/bP (**2**) and Ni/bP (**3**) devices in dry conditions.

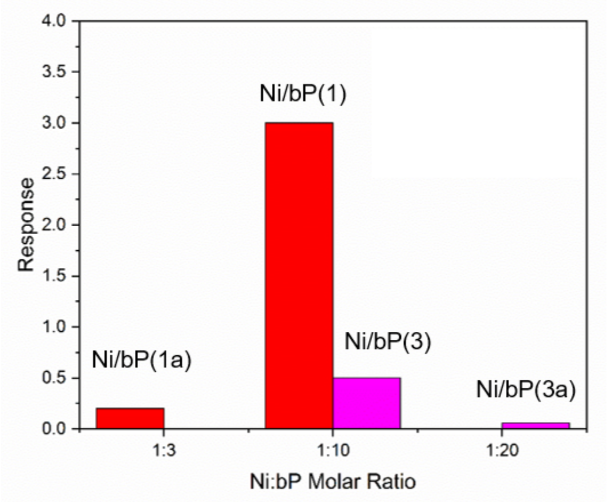

Figure S13. Variation of the electrical response with different amount of Ni NPs on bP surface to 1 ppm of NO_2 in dry conditions.

References

(1) Lange, S.; Schmidt, P.; Nilges, T. Au 3 SnP 7 @Black Phosphorus: An Easy Access to Black Phosphorus. *Inorg. Chem.* **2007**, *46* (10), 4028–4035. https://doi.org/10.1021/ic062192q.

(2) Hu, T.; Hong, J. First-Principles Study of Metal Adatom Adsorption on Black Phosphorene. *J. Phys. Chem. C* **2015**, *119* (15), 8199–8207. https://doi.org/10.1021/acs.jpcc.5b01300.

(3) Phuc, H. V.; Hieu, N. N.; Ilyasov, V. V.; Phuong, L. T. T.; Nguyen, C. V. First Principles Study of the Electronic Properties and Band Gap Modulation of Two-Dimensional Phosphorene Monolayer: Effect of Strain Engineering. *Superlattices Microstruct.* **2018**, *118*, 289–297. https://doi.org/10.1016/j.spmi.2018.04.018.

(4) Wei, Q.; Peng, X. Superior Mechanical Flexibility of Phosphorene and Few-Layer Black Phosphorus. *Appl. Phys. Lett.* **2014**, *104* (25), 251915. https://doi.org/10.1063/1.4885215.

(5) Speranza, G.; Canteri, R. RxpsG a New Open Project for Photoelectron and Electron Spectroscopy Data Processing. *SoftwareX* **2019**, *10*, 100282. https://doi.org/10.1016/j.softx.2019.100282.
